# Maternal Roughage Sources Influence the Gastrointestinal Development of Goat Kids by Modulating the Colonization of Gastrointestinal Microbiota

**DOI:** 10.3390/ani15030393

**Published:** 2025-01-30

**Authors:** Haidong Du, Kenan Li, Wenliang Guo, Meila Na, Jing Zhang, Renhua Na

**Affiliations:** 1 College of Animal Science, Inner Mongolia Agricultural University, Hohhot 010018, China; duhaidong1110@163.com (H.D.); 18686197338@163.com (W.G.); 15548710467@163.com (M.N.); zhangjing230518@163.com (J.Z.); 2 Grassland Research Institute of Chinese Academy of Agricultural Sciences, Hohhot 010010, China; likenan0826@yeah.net

**Keywords:** maternal diet, microbiome, metabolome, transcriptome, vertical transmission, gastrointestinal development, immune function, goats

## Abstract

During pregnancy and lactation, maternal nutrition is linked to the full development of offspring and may have long-term or lifelong effects. However, the influence of the doe’s diet on the gastrointestinal (GI) tract of young kids remains largely unexplored. Therefore, we investigated the effects of doe roughage sources (alfalfa hay, AH, or corn straw, CS) during pregnancy and lactation on kid growth, GI morphology, barrier function, metabolism, immunity, and microbiome composition. The results indicate that, compared with the CS group, does fed an AH diet had significantly higher feed intake (*p* < 0.01). However, CS-fed does exhibited higher neutral detergent fiber (NDF) digestibility (*p* < 0.05). There were no significant differences in animal (doe or kid) weight among the groups (*p* > 0.05). In the rumen of goat kids, the AH group exhibited a higher papillae width and increased levels of interleukin-10 (IL-10) compared with the CS group (*p* < 0.05). In the jejunum of goat kids, the AH group showed a higher villus-height-to-crypt-depth (VH/CD) ratio, as well as elevated levels of secretory immunoglobulin A (SIgA), immunoglobulin G (IgG), IL-10, acetate, and total volatile fatty acids (TVFAs), when compared with the CS group (*p* < 0.05). Transcriptome analysis revealed that the source of roughage in does was associated with changes in the GI transcriptome of the kids. Differentially expressed genes (DEGs) in the rumen were mainly associated with tissue development and immune regulation, while the DEGs in the jejunum were mainly associated with the regulation of transferase activity. Spearman correlation analyses indicated significant associations between GI DEGs and phenotypic indicators related to GI development, immunity, and metabolism. LEfSe analysis identified 14 rumen microbial biomarkers and 6 jejunum microbial biomarkers. Notably, these microorganisms were also enriched in the rumen or day 28 milk of the does. Further microbial composition analysis revealed significant correlations between the rumen and milk microbiomes of does and the rumen or jejunum microbiomes of kids. Association analyses indicated that microbial biomarkers interact with host genes, thereby affecting the development and function of the GI system. Additionally, correlation analyses revealed significant association between milk metabolites and the rumen and jejunum microbiomes of kids. This study demonstrated that maternal diet significantly influences the development of microbial ecosystems in offspring by modulating microbial communities and metabolite composition. The early colonization of GI microorganisms is crucial for the structural development, barrier function, immune capacity, and microbial metabolic activity of the GI system.

## Simple Summary

The juvenile period represents a critical stage in the development of ruminants, with significant implications for their subsequent growth and physiological functions. During this phase, young animals are at an elevated risk of mortality due to the immaturity of their immune and digestive systems. The nutritional status of the dam is of paramount importance to the health and growth of the offspring, as it directly influences the availability of nutrients to the young animal, which, in turn, affects growth rate, immune development, and overall health status. This study investigates the impact of maternal diet during pregnancy and lactation on goat kid growth, gastrointestinal health, and microbiome composition. We found that goat does who were fed alfalfa hay, as opposed to corn straw, produced kids with superior growth, improved gastrointestinal morphology, enhanced immune function, and a more efficient metabolism. These outcomes were associated with changes in the kids’ gastrointestinal microbiome and gene expression. The findings suggest that optimizing maternal nutrition can significantly enhance kid health, offering potential benefits for the livestock industry by promoting growth, immune responses, and overall productivity, which are crucial for improving animal performance and health.

## 1. Introduction

Ruminants are a substantial component of animal husbandry, providing large amounts of high-quality meat and dairy products to society. The juvenile period is critical for ruminant development, as nutritional intake and health status during this stage decisively influence subsequent growth. However, it is also the period with the highest mortality rates [1]. In extensive pastoral livestock systems, approximately 11–39% of singleton lamb and 21–38% of multiple-born lamb die before pre-weaning [2]. In goat farms, the mortality rate for young goat ranges from 16% to 33% [3]. The primary factors contributing to this high mortality include low birth weight and various infectious diseases, such as pneumonia, diarrhea/enteritis, and sepsis [3,4]. Therefore, ensuring balanced nutrition for pregnant goats during gestation is crucial, as it directly influences the birth weight and lays the foundation for preventing subsequent health issues [3]. Furthermore, establishing a stable and beneficial symbiotic microbial community early in the kid’s life, along with promoting the development of gastrointestinal functions, is essential in significantly improving survival rates, preventing gastrointestinal infections, and enhancing overall health [5,6,7]. The gastrointestinal (GI) tract is fundamental to the health and development of mammals, influencing nutrient absorption, digestion, immune function, and metabolic homeostasis. The health and function of the GI tract depend on complex interactions between the host and its microbial communities, particularly in the early stages of life [5]. Early microbial colonization in the GI tract is essential for establishing a functional and balanced microbiota, which supports gastrointestinal health, growth, and immune system maturation [6,7,8]. The rumen serves as the primary site for microbial fermentation, nutrient absorption, and metabolism; thus, its developmental status affects the health and growth of ruminants. In early life, the rumen is not fully developed, characterized by a smooth inner wall and the absence of the characteristic papillae, which limits its ability to digest complex nutrients [9]. Consequently, promoting the functional development of the rumen and ensuring the establishment and efficient operation of microbial communities has become a priority and critical challenge in the care of young ruminants. At this stage, fermentation in the rumen of pre-ruminant animals is minimal, and young ruminants primarily rely on intestinal digestion to meet their energy and nutritional needs [10]. The small intestine of ruminants is functional at birth [11]. For a more efficient response to increased nutrient absorption demands, the gut tissues continue to develop and show rapid adaptability to the significant increase in nutrient levels during lactation [11]. Simultaneously, microorganisms in the gut colonize rapidly after birth and play an essential role in supporting gut development [12]. During lactation, the initial establishment of gastrointestinal microbial communities in young animals plays a pivotal role in their overall developmental programming [13].

The dam diet plays a critical factor in shaping the GI microbial communities of young animals [14,15]. During the neonatal stage, the composition of the GI microbiota undergoes significant fluctuations, demonstrating its high adaptability and plasticity [16]. This provides a valuable opportunity to optimize offspring microbiomes through the regulation of the maternal diet, potentially leading to significant improvements in both short-term and long-term health outcomes for young animals. Roughage is an indispensable feed source for ruminants, and maintaining adequate roughage intake is essential for their health and survival. Different types of roughage have distinct physical structures, chemical compositions, and nutritional compositions, which can differentially impact the microbial composition and metabolic processes in ruminants [17,18]. Despite the crucial role of roughage in ruminant nutrition, relatively few studies have been conducted on the impact of roughage sources for goat does on their offspring. Existing research primarily focuses on how the doe’s roughage influences the growth performance of their kids [19,20,21]. The impact of goat doe’s roughage sources on the GI development and health of offspring remains understudied. We hypothesize that feeding different types of roughage to goat does during pregnancy and lactation may lead to varying effects on the growth, health, microbial colonization, and GI development of their offspring. In northern China, alfalfa and corn are widely cultivated, and their dried forage products serve as essential feed for livestock [22,23]. In Inner Mongolia, corn straw and alfalfa hay are commonly used as roughage in cashmere goat farming. Therefore, we selected these two types of roughage as the primary feed source for goat does to investigate the effects of maternal roughage sources during gestation and lactation on the microbial colonization, GI morphology, barrier function, metabolism, and immunity in offspring kids. This study aims to determine whether the dam’s diet influences the gastrointestinal development of offspring during the early developmental window.

## 2. Materials and Methods

### 2.1. Experimental Design

The animal experiment was performed at the Inner Mongolia Agricultural University experimental animal farm (Hohhot, Inner Mongolia, China). Prior to the trial, 180 does were bred by artificial insemination after induced estrus. All does in this study received frozen semen from the same male goats. At 59 days post artificial insemination, the pregnancy status was checked by abdominal ultrasonography, and a single or twin pregnancy was recorded. Before the feeding trial began, the does were fed with the diets used by the experimental animal farm (the does were fed corn straw ad libitum, and 50 g commercial concentrate per doe per day). On the 60th day of gestation, 36 pregnant cashmere goats of the same age (4 years old) and similar weight (46.22 ± 2.75 kg) were selected for the study. The pregnant does were randomly assigned to two treatment groups. Each treatment group consisted of 18 does, including 8 with twin pregnancies and 10 with single pregnancies. There were three experimental replicates per treatment group, and 6 does per replicate. Six does per pen were placed in covered dirt pens with automatic watering systems. The experimental enclosure consisted of a feeding area and a resting area, separated by a manually operated door. The floor of the enclosure was entirely covered with dirt, and no bedding was provided. A separate pen was constructed in the resting area to house the kids. During the experimental period, the does were fed either a corn straw-based diet (CS group; *n* = 18) or an alfalfa hay-based diet (AH group; *n* = 18), with free access to drinking water. The does were fed twice daily, at 8 a.m. and 4 p.m., from the start of the trial until seven days postpartum. Both does and kids were free to move in the feeding and rest areas, and the does were free to feed. From eight days postpartum to the end of the trial, the does and kids were primarily active in the resting area. The does were permitted access to the feeding area from 8 a.m. to 10 a.m. and from 4 p.m. to 6 p.m. each day. During the does’ feeding periods, the kids were kept in a separate enclosure within the resting area to prevent contact with the dams’ diet. Once the does had finished feeding, they returned to the playground and the kids were released. The AH and CS diets were isonitrogenous and isoenergetic, meeting or exceeding the nutrient requirements of cashmere goats (NY/T 4048-2021) [24]. The dietary composition is presented in Table S1. After parturition (the average gestation duration for does was 148 days in the CS group and 149 days in the AH group), the kids were housed in the same enclosure as their respective does, ensuring that milk served as their sole dietary source throughout the trial. Furthermore, for does that give birth to twins, one kid was immediately removed after birth to ensure that each doe was responsible for nurturing only a single kid. Thirty-six doe–kid pairs were used in the feeding trial. The CS group had 11 male kids and 7 female kids, and the AH group had 8 male kids and 10 female kids. The feed intake of the does was calculated by recording the amount of feed offered and residue. The kids were weighed every 7 days, and the occurrence of diarrhea was recorded daily. During the trial, one kid from each group died shortly after birth; however, no kids exhibited diarrhea.

### 2.2. Sample Collection

On postnatal day 28, after the kids had finished suckling, six does (selected before morning feeding) were randomly chosen from each group for sampling. Approximately 50 mL of rumen fluid was collected from each doe using a vacuum sampler, subsequently filtered through four layers of gauze, and then placed into a cryopreservation tube. The udder of each doe was wiped with alcohol-soaked cotton and sterile gauze, followed by manual milk collection. Both the rumen fluid and milk samples were stored at −80 °C for subsequent microbiological analysis and non-targeted metabolomics analysis. At 28 d of age, 2 kids from each replicate group were chosen based on the average body weight. A total of 12 kids from both treatment groups were selected for sampling. After 3 h of morning feeding, the ranch veterinarian euthanized the kid with an intravenous overdose of sodium pentobarbital (50 mg/kg BW), then exsanguination under anesthesia. The contents of the rumen and jejunum were collected and stored at −80 °C for microbiological analysis. Tissue samples were obtained from the ventral sac region of the rumen and the middle segment (approximately 650 cm from abomasum sphincter) of the jejunum. One portion of the tissue was fixed in 4% paraformaldehyde for morphological analysis and immunofluorescence staining, while the remainder was washed with phosphate buffered saline (PBS) and stored at −80 °C for biochemical assessment and transcriptomic analysis.

### 2.3. Nutrient Digestibility

A digestibility trial was conducted using the acid-insoluble ash (AIA) method [25]. Briefly, ewe diets and fecal samples collected during the experiment were dried at 65 °C, then ground and sieved through a 1 mm screen. The resulting samples were then utilized for chemical analysis, and the digestibility values of the nutritional components were calculated using the following formula: X digestibility = [1 − (diet AIA content/fecal AIA content) × (fecal X content/diet X content)] × 100%, X = neutral detergent fiber (NDF), acid detergent fiber (ADF), crude protein and crude fat.

### 2.4. Histomorphometric Analysis

Histomorphometric analysis was adapted from the method of Shang et al. [8]. Twelve kids (*n* = 6/group) were sacrificed for histomorphometric analysis, and 24 tissue samples were collected (12 from the jejunum and 12 from the rumen). Rumen and jejunum tissues were fixed in 4% paraformaldehyde, dehydrated, and embedded in paraffin. Sections were cut at a 5 μm thickness (three non-continuous slices were selected from each sample) and stained with hematoxylin and eosin. A total of three slices were obtained for each tissue, with one image taken per section. Ten morphologically intact structures (e.g., rumen papillae, rumen muscularis, jejunal villi, crypts, or jejunal muscularis) were selected from the three slices using an Olympus SZX 10 microscope (Olympus Corporation, Tokyo, Japan), and measurements were taken using the Image-Pro System Plus 6.0 (MEDIA CYBERNETICS, Bethesda, MD, USA). The measured parameters included the length of rumen papillae (from the tip to the base), the width of rumen papillae (average width at the base, middle, and tip), the thickness of rumen muscularis (from the inner muscularis to the muscularis externa), the height of jejunal villi (from the tip to the base), the depth of crypts (from the crypt base to the top opening), and the thickness of jejunal muscularis (from the muscularis externa to the crypt base). The final result was determined by calculating the mean of the 10 measurements. During data collection, the experimenter was blinded to group allocation.

### 2.5. Immune Parameters Determination

A total of 0.5 g either of rumen or jejunum tissue was placed into a test tube, followed by the addition of 4.5 mL of saline. The tissue samples were then homogenized using a tissue homogenizer. The homogenates underwent centrifugation at 3000× *g* for 15 min at 4 °C, and the supernatant was collected for biochemical analysis. Commercial goat ELISA kits (Shanghai Enzyme-linked Biotechnology Co., Ltd., Shanghai, China) were used to determine the levels of secretory immunoglobulin A (SIgA), immunoglobulin G (IgG), immunoglobulin M (IgM), as well as the cytokine levels of the proinflammatory factors interleukin-6 (IL-6) and tumor necrosis factor-α (TNF-α), and the anti-inflammatory factor interleukin-10 (IL-10). All procedures were performed according to the manufacturer’s instructions. The assay parameters were intra-assay CV < 9%, and inter-assay CV < 15%.

### 2.6. Volatile Fatty Acids Determination

Rumen fluid was combined with 25% metaphosphoric acid in a 5:1 ratio and then subjected to centrifugation at 10,000× *g* for 15 min at 4 °C. The resulting fluid was filtered through a 0.22 μm aqueous filter membrane, and the supernatant was subsequently analyzed using a gas chromatograph (Clarus680, PerkinElmer, Waltham, MA, USA) for the determination of VFAs [26].

### 2.7. Immunofluorescent Staining

Immunofluorescent staining was performed by Wuhan Servicebio Biological Technology, Ltd. (Wuhan, China). Paraffin sections of the rumen and jejunum were dewaxed in water, and antigen retrieval was performed using EDTA, followed by blocking with 10% donkey serum. The primary antibodies—rabbit anti-tight junction protein ZO-1 (ZO-1, Cat No. GB111402, 1:500 dilution), rabbit anti-claudin-1 (claudin-1, Cat No. GB112543, 1:500 dilution), and rabbit anti-Mucin2 (MUC2, Cat No. GB11344-100, 1:500 dilution)—were then applied and incubated overnight at 4 °C. The slides were incubated for 50 min with secondary antibodies, goat anti-rabbit antibodies (Cat No. GB23303, 1:3000 dilution), followed by treatment with 0.5 μg/mL DAPI for 10 min. Images were captured using confocal laser microscopy. Quantitative analysis of claudin-1, ZO-1, and MUC2 molecules was performed using ImageJ software (version 2.0.0).

### 2.8. Microbiota Analysis

Microbial genomic DNA was extracted from rumen, jejunum, and milk samples using the Magnetic Soil and Stool DNA Kit (TianGen, BeiJing, China, Catalog #: DP712). DNA integrity was determined by 1% agarose gel electrophoresis. The V4 region of the bacterial 16S rRNA gene was amplified by PCR with universal primers (515F and 806R), as previously described [27]. Amplicon library sequencing was performed on an Illumina NovaSeq 6000 platform (Illumina, Beijing, China). Raw sequencing reads were clipped and quality filtered using flash (v1.2.11) and fastp (v0.23.1) software. QIIME2 (Version QiiME2-202202) was employed to remove chimera sequences and obtain amplicon sequence variants (ASVs), which were annotated using the Silva (v138) database.

QIIME2 software was used to measure the Chao 1 index. Non-metric multidimensional scaling (NMDS) analysis based on Bray–Curtis distance was conducted with R software (version 3.6.1) and complemented with analysis of similarity (ANOSIM). LEfSe analysis was employed to identify microbial biomarkers among groups (LDA > 2.5, *p* < 0.05). Source Tracker software (https://cloud.majorbio.com/page/tools/, accessed on 11 December 2024) was used to predict potential sources of the rumen and jejunum microbiota in kids.

### 2.9. Transcriptome Analysis

Total RNA was extracted from the rumen and jejunum tissues of kids using the Trizol reagent kit (Invitrogen, Carlsbad, CA, USA). RNA quality and concentration were detected via agarose gel electrophoresis and a Nanodrop ultra-UV spectrophotometer, while molecular integrity was analyzed using an Agilent 2100 Bioanalyzer (Agilent Technologies, Santa Clara, CA, USA). A library was constructed with the Illumina TruSeq kit (Illumina, San Diego, CA, USA), enriching mRNA with oligo(dT) magnetic beads. The library preparations were sequenced on an Illumina NovaSeq platform (Illumina, Beijing, China), generating 150 bp paired-end reads. Adapter sequences and low-quality reads were removed using fastp (v0.20.0). Clean reads were aligned to the reference cashmere goat genome (ensembl_capra_hircus_ars1_gca_001704415_1) using HISAT2 (v2.0.5), and gene expression levels were calculated with RSEM (v1.2.12). The mapping efficiency varied from 85.18~89.85%. DEGs were identified using DESeq software (DESeq version 1.39.0) with thresholds of *Padj* < 0.05 and log2|Fold Change| > 1. GO and KEGG enrichment analyses were performed using clusterProfiler (v3.8.1).

### 2.10. Metabolomic Profiling

A total of 100 µL of milk was added to a 1.5 mL centrifuge tube containing 400 µL of a 1:1 (*v*:*v*) acetonitrile: methanol solution with 0.02 mg/mL internal standard (L-2-chlorophenylalanine) for metabolite extraction. Samples were vortexed for 30 s, sonicated at 5 °C for 30 min (40 kHz), and then placed at −20 °C for 30 min to precipitate proteins. Following centrifugation at 4 °C for 15 min (13,000× *g*), the supernatant was removed and evaporated under nitrogen. The residue was re-solubilized in 100 µL of a 1:1 (*v*:*v*) acetonitrile: water solution, sonicated for 5 min, and centrifuged again (13,000× *g*, 4 °C for 10 min). The supernatant was collected for LC-MS/MS analysis.

Quality control samples were prepared by mixing equal amounts of all samples. LC-MS/MS analysis was performed using a Thermo UHPLC-Q Exactive system with an ACQUITY HSS T3 column (100 mm × 2.1 mm i.d., 1.8 μm; Waters Corporation, Milford, MA, USA) at Majorbio Bio-Pharm Technology Co. Ltd. (Shanghai, China). Raw data were deconvolved using Progenesis QI (Waters Corporation, Milford, MA, USA), and metabolites were annotated with HMDB (http://www.hmdb.ca/, accessed on 11 December 2024), Metlin (https://metlin.scripps.edu/, accessed on 11 December 2024) and the Majorbio database. The R package “ropls” (Version 1.6.2) conducted orthogonal partial least squares discriminant analysis (OPLS-DA) with 7-cycle interactive validation for model stability. Metabolites with VIP > 1 and *p* < 0.05 were considered significantly different, based on OPLS-DA results. Differential metabolites were mapped to biochemical pathways using KEGG pathway analysis (http://www.genome.jp/kegg/, accessed on 11 December 2024).

### 2.11. Statistical Analysis

Statistical comparisons were carried out using Student’s *t*-test or Welch’s *t*-test (SAS software, v.9.2, SAS Institute, Cary, NC, USA). Data are shown as mean ± standard deviation (STDEV). Spearman test and Procrustes test were used for the correlation analysis. *p* value ≤ 0.05 was considered statistically significant for all comparisons.

## 3. Results

### 3.1. The Impact of the Doe’s Diet on Her Feed Intake, Body Weight, and the Body Weight of Her Kid

**Figure 1:**
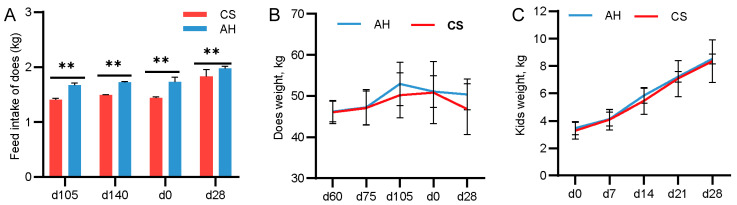
Effect of the maternal diet on offspring growth. (**A**) The doe daily feed intake on days 105 and 140 of gestion and days 0 and 28 of lactation. *n* = 18/group. (**B**) The doe weight on days 60, 75 and 105 of gestation and days 0 and 28 of lactation. *n* = 18/group. (**C**) The kid weight on days 0, 7, 14, 21 and 28 of lactation. *n* = 36. CS, corn straw group. AH, Alfalfa hay group. Data are shown as mean ± STDEV. ** *p*  <  0.01.

From day 105 of gestation to day 28 of lactation, does fed the AH diet exhibited significantly higher feed intake compared with those fed the CS diet (*p* < 0.01; Figure 1A). Both does and kids in the AH group had greater body weights than those in the CS group; however, this difference was not statistically significant.

### 3.2. Effects of Roughage Sources on Doe Apparent Nutrient Digestibility

As shown in Table 1, during gestation, the digestibility of NDF and ADF in the CS group does was significantly higher than in the AH group (*p* < 0.05). During lactation, the digestibility of NDF remained significantly higher in the CS group does compared with the AH group (*p* < 0.01).

### 3.3. Effect of the Maternal Diet on Offspring Gastrointestinal Morphology

**Figure 2:**
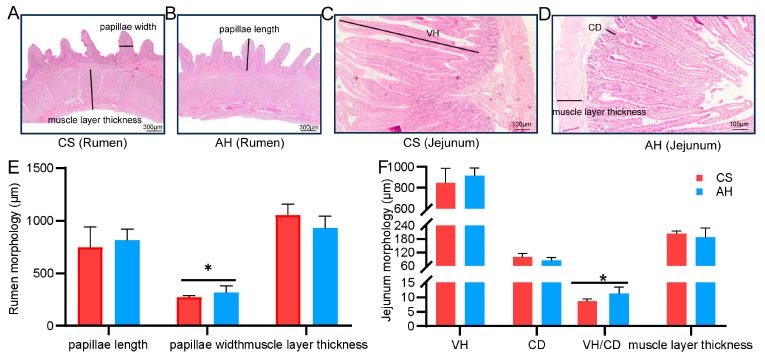
Effect of the maternal diet on offspring gastrointestinal morphology. Comparisons of the rumen (**A**,**B**,**E**) and jejunum (**C**,**D**,**F**) morphology between the two groups. VH, villus height. CD, crypt depth. Data are shown as mean ± STDEV. * *p*  <  0.05. *n* = 6/group.

To investigate the impact of doe diet on kid GI development, we compared the morphological characteristics of jejunum and rumen tissues between kids in the AH and CS groups (Figure 2). Compared with the CS group, the papillae width of the rumen and the villus height: crypt depth (VH/CD) ratio of the jejunum were significantly greater in the AH group (*p* < 0.05; Figure 2E,F). Rumen muscle layer thickness was numerically greater in the CS group than in the AH group (Figure 2E).

### 3.4. Effect of the Maternal Diet on Offspring Gastrointestinal Immunity

**Figure 3:**
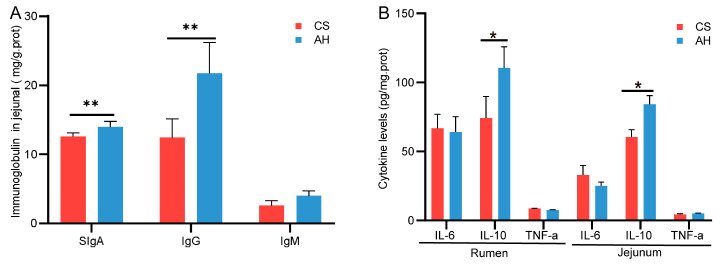
Effect of the maternal diet on offspring gastrointestinal immunity. (**A**) Comparison of the differences in intestinal immunoglobulin between the two groups. (**B**) Comparison of the differences in immune factors in the rumen and jejunum between the groups. CS, corn straw group. AH, alfalfa hay group. Data are shown as mean ± STDEV. * *p*  <  0.05, ** *p*  <  0.01. *n* = 6/group.

In terms of immune indicators, kids from AH-fed does had higher levels of jejunum SIgA and IgG (Figure 3A), as well as higher levels of rumen and jejunum IL-10 (Figure 3B), compared with the CS group (*p* < 0.05).

### 3.5. Effect of the Maternal Diet on Offspring Gastrointestinal Volatile Fatty Acids

**Figure 4:**
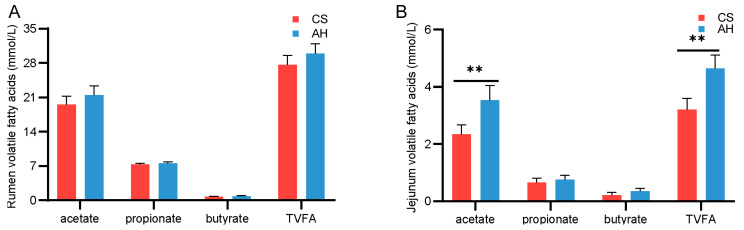
Effect of the maternal diet on offspring gastrointestinal volatile fatty acids. (**A**) Comparison of the differences in VFAs in the rumen (**A**) and jejunum (**B**) between the groups. TVFA, total volatile fatty acids. CS, corn straw group. AH, alfalfa hay group. Data are shown as mean ± STDEV. ** *p*  <  0.01. *n* = 6/group.

Regarding metabolic indicators, the AH group had higher levels of TVFA and acetate in the jejunum compared with the CS group (*p* < 0.01; Figure 4B), while levels of propionate and butyrate remained unchanged (*p* > 0.05; Figure 4B). However, the concentrations of VFAs in the rumen were unaffected by the maternal diet (Figure 4A).

### 3.6. Effect of the Maternal Diet on Offspring Gastrointestinal Epithelial Barrier Integrity

**Figure 5:**
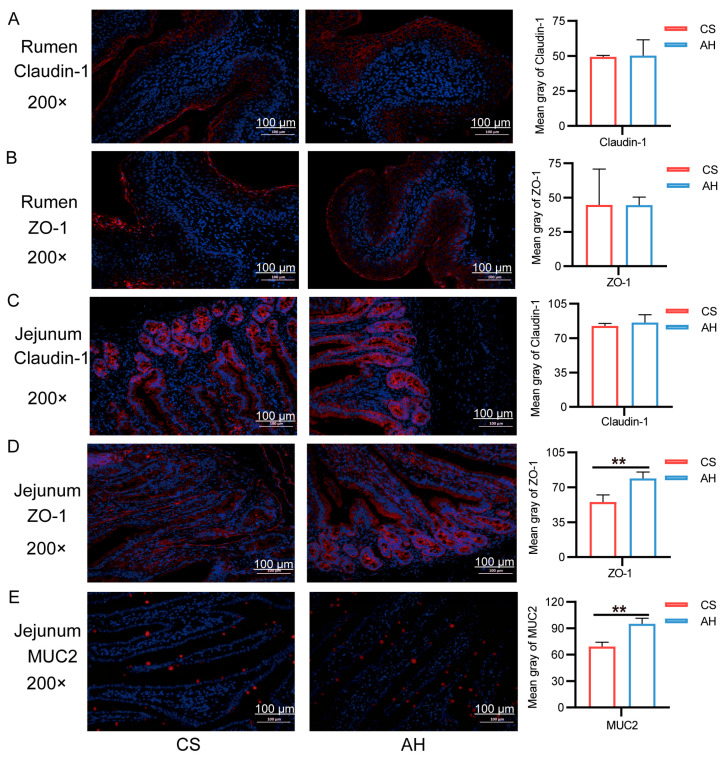
Effect of the maternal diet on offspring gastrointestinal epithelial barrier integrity. Immunofluorescence images showing protein expression of rumen claudin-1 (**A**) and ZO-1 (**B**). Immunofluorescence images showing protein expression of jejunum claudin-1 (**C**) and ZO-1 (**D**) and MUC2 (**E**). Red, target proteins. Blue, DAPI-stained nuclei. CS, corn straw group. AH, alfalfa hay group. Data are shown as mean ± STDEV. ** *p*  <  0.01. On the left is the CS group picture, on the right is the AH group picture. *n* = 6/group.

Immunofluorescence staining of claudin-1 and ZO-1 in the rumen, as well as claudin-1, ZO-1, and MUC2 in the jejunum, was performed to determine the distribution and expression of barrier proteins (Figure 5). We found that ZO-1 and MUC2 expression in the jejunum was higher in the AH group than in the CS group (*p* < 0.01; Figure 5D,E). However, maternal diet sources did not affect the expression of ZO-1 and claudin-1 in the rumen of kids (*p* > 0.05; Figure 5A,B).

### 3.7. Effect of the Maternal Diet on Offspring Gastrointestinal Transcriptome

**Figure 6:**
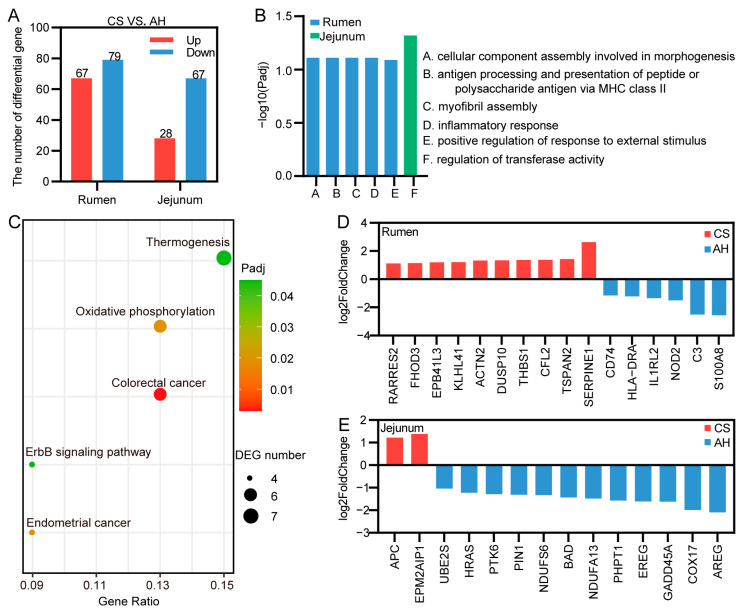
Effect of the maternal diet on offspring gastrointestinal transcriptome. (**A**) Histogram of differentially expressed genes (DEGs). (**B**) Plot shows the significantly enriched GO biological process terms in the rumen and jejunum (*Padj* < 0.1). (**C**) Scatter diagram shows the significantly enriched KEGG pathways in kid jejunum (*Padj* < 0.05). Characterization of DEGs involved in significantly enriched pathways in the rumen (**D**) and jejunum (**E**). CS, corn straw group. AH, alfalfa hay group. *n* = 6/group.

To identify differences in gene expression levels between the two groups, RNA-seq transcriptome sequencing was performed (Figure 6). In the kid rumen, compared with the AH group, 67 DEGs were found to be upregulated, and 79 DEGs were downregulated in the CS group (Figure 6A). In kid jejunum, compared with the AH group, 28 DEGs were upregulated, and 67 DEGs were downregulated in the CS group (Figure 6A). Subsequently, GO enrichment analysis (biological processes) and KEGG pathway enrichment analysis were performed for the DEGs (Figure 6B). The enriched GO terms of rumen were mainly associated with cell and structural development processes (A, C; *Padj* < 0.1; Figure 6B), immune response, and response to stimulus (B, D, E; *Padj* < 0.1; Figure 6B). No significant enrichment of KEGG pathways was observed in the rumen. In the jejunum, the most significantly enriched GO term was regulation of transferase activity (F; *Padj* < 0.1; Figure 6B). The KEGG enrichment analysis indicated notable enrichment of DEGs in the oxidative phosphorylation, thermogenesis, and ErbB signaling pathways (Figure 6C).

The genes in the enriched GO entries (*Padj* < 0.1) and enriched KEGG pathways (*Padj* < 0.05) were selected as candidate genes (Table S2). In the rumen, there were 16 candidate genes, of which 10 were upregulated in the CS group and 6 in the AH group (Figure 6D). In the jejunum, the respective numbers were 2 and 12 for the CS and AH groups (Figure 6E).

### 3.8. Effect of the Maternal Diet on Offspring Gastrointestinal Microbiota

**Figure 7:**
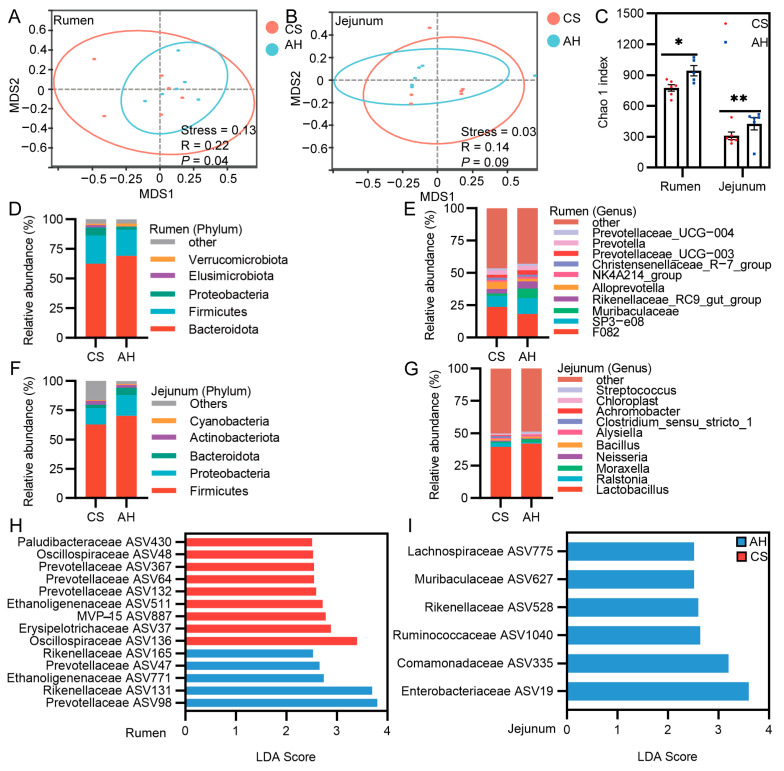
Effect of the maternal diet on offspring gastrointestinal microbiota. NMDS analysis of rumen (**A**) and jejunum (**B**) microbiome composition was performed using Bray–Curtis distance, and statistical analysis using ANOSIM analysis. (**C**) α diversity-Chao1 indexes of the rumen and jejunum microbiota. Rumen microbial composition at the level of phylum (**D**) and genus (**E**). Jejunum microbial composition at the level of phylum (**F**) and genus (**G**). LEfSe analyses were used to identify the rumen (**H**) and jejunum (**I**) microbial community structure (LDA > 2.5). CS, corn straw group. AH, alfalfa hay group. Red and blue circles, 95% confidence interval. * *p*  <  0.05, ** *p*  <  0.01. *n* = 6/group.

According to NMDS and ANOSIM analysis, the two groups of kids had significantly separated in rumen (Stress = 0.13, ANOSIM, R = 0.22, *p* = 0.04) or jejunum (Stress = 0.03, ANOSIM, R = 0.14, *p* = 0.09) flora structure (Figure 7A,B). Compared with the CS group, the Chao1 index for microbial richness in both the rumen and jejunum was significantly higher in the AH group (*p* < 0.05; Figure 7C), indicating that maternal diet significantly influences the microbial diversity of offspring. The community composition at both the phylum and genus levels was then summarized for the AH and CS groups. Firmicutes, Bacteroidetes, and Proteobacteria were the dominant phyla in both the rumen and jejunum (Figure 7D,F). In the rumen, the relative abundance of Bacteroidetes was greater in the AH group (69.10%) compared with the CS group (62.47%), while Firmicutes was more abundant in the CS group (23.71%) than in the AH group (21.87%; Table S3). In the jejunum, the AH group exhibited higher relative abundances of Firmicutes (AH vs. CS, 70.08% vs. 62.75%) and Proteobacteria (AH vs. CS, 17.79% vs. 14.02%; Table S3). At the genus level, *F082* and *SP3-e08* were the dominant genera in the rumen (Figure 7E). *F082* had the highest relative abundance (23.59%) in the CS group, while *F082* and *SP3-e08* had the highest relative abundance (18.11% and 12.34%, respectively; Table S3) in the AH group. In the jejunum, *Lactobacillus* was the most abundant genus (Figure 7G), with a higher abundance observed in the AH group (AH vs. CS, 42.08% vs. 39.53%; Table S4). LEfSe analysis identified 14 differentially abundant taxa in the rumen, with 5 taxa enriched in the AH group and 9 taxa in the CS group. Specifically, *Paludibacteraceae* (ASV430), *Oscillospiraceae* (ASV48, ASV136) *Prevotellaceae* (ASV367, ASV64, ASV132) *Ethanoligenenaceae* (ASV511), *MVP-15* (ASV887), and *Erysipelotrichaceae* (ASV37) were more abundant in the CS group, while the relative abundance of *Prevotellaceae* (ASV47, ASV98), *Rikenellaceae* (ASV131, ASV165) and *Ethanoligenenaceae* (ASV771) were significantly higher in the AH group (Figure 7H). In the jejunum, the AH group had significantly higher relative abundances of *Enterobacteriaceae* (ASV19), *Comamonadaceae* (ASV335), *Ruminococcaceae* (ASV1040), *Rikenellaceae* (ASV528), *Muribaculaceae* (ASV627), and *Lachnospiraceae* (ASV775) compared with the CS group (Figure 7I).

### 3.9. The Vertical Mother-to-Infant Microbial Transmission

**Figure 8:**
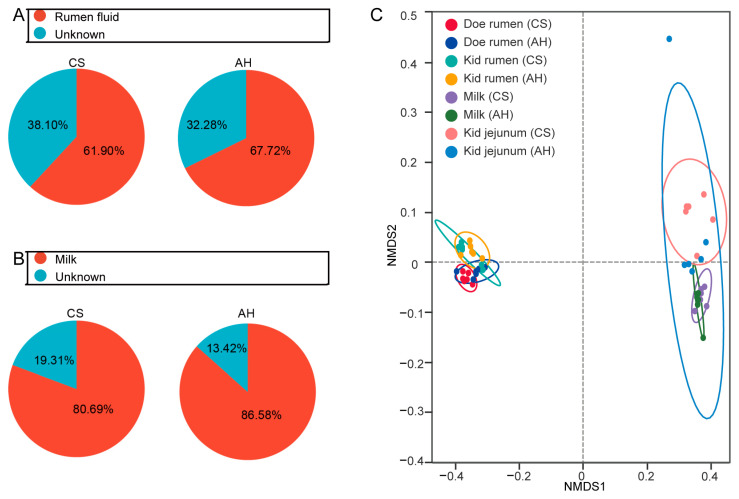
The vertical mother-to-infant microbial transmission. (**A**) SourceTracker was used to estimate the proportions of microbes in the kid rumen from the doe rumen. (**B**) Source of the milk proportions of microbes in the kid jejunum estimated using SourceTracker. (**C**) NMDS analysis compared the β diversity of the microbiota of does and kids. Circles, 95% confidence interval. CS, corn straw group. AH, alfalfa hay group. *n* = 12/group.

To evaluate the similarity of the kid rumen and jejunal microbiota to that of the doe rumen and milk microbiota, we used SourceTracker analyses and NMDS (Figure 8). In the CS group, 61.90% of the kid rumen microbiota originated from doe rumen fluid (Figure 8A); while the kid jejunum received 80.69% of its bacteria from milk (Figure 8B). In the AH group, 67.72% of the microbes in kid rumen were sourced from doe rumen fluid (Figure 8A), and 86.58% of the microbes in the kid jejunum originated from milk (Figure 8B). The NMDS plot revealed two distinct clusters: the rumen samples of both does and kids grouped together, while the kid jejunum samples clustered with the milk samples (Figure 8C). We further analyzed the correlation between doe differential microbes and kid differential microbes using Procrustes analysis (Figure S1). The result shows a significant correlation between differential ASVs in the rumen of does and kids (*p*  =  0.002, M^2^ = 0.81). Similarly, a significant correlation was also observed between jejunum differential ASVs and milk differential ASVs (*p*  =  0.002, M^2^ = 0.33). The above results reveal that maternal microbes are transferred to the offspring rumen and gut, influencing the initial establishment of the offspring microbiota. Furthermore, the dietary sources of the doe are critical in shaping the microbiota composition in both does and kids.

### 3.10. The Host Microbiota Is Correlated with the Host Genes and Gastrointestinal Phenotype Data

**Figure 9:**
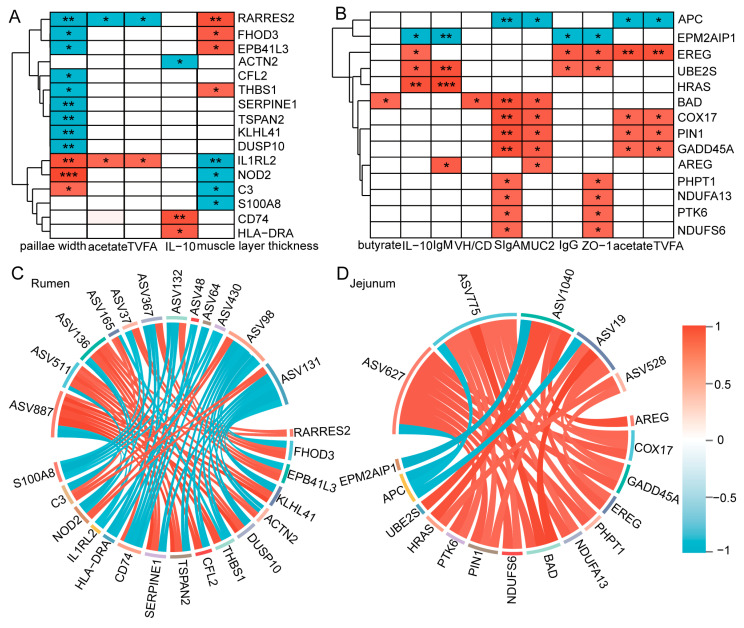
The host microbiota is correlated with the host genes and gastrointestinal phenotype data. Spearman correlations between DEGs and phenotype data in the rumen (**A**) and jejunum (**B**). Spearman correlations between DEGs and microbial markers in the rumen (**C**) and jejunum (**D**). Significant correlation was defined as |r| > 0.8 and *p* < 0.05. * *p*  <  0.05, ** *p*  <  0.01. *** *p*  <  0.001. Red, positive correlation; blue, negative correlation. *n* = 6/group.

We first investigated the correlation between candidate genes and GI phenotype data including immunity, metabolism, morphology, and structure. As shown in Figure 9A, the papillae width showed the greatest number of significant correlations with candidate genes (*n* = 12), followed by muscle layer thickness (*n* = 8), and IL-10 (*n* = 3). Furthermore, certain genes associated with “immune response and response to stimulus” were positively correlated with papillae width, IL-10, acetate and TVFA. Muscle layer thickness was significantly correlated with genes related to “cell and structural development processes.” In the jejunum (Figure 9B), immune indicators (IL-10, IgM, SIgA), metabolic indicators (acetate, TVFA), and tissue morphology and structural indicators (VH/CD, MUC2, ZO-1) were significantly correlated with most candidate genes. Notably, these phenotypic data exhibited positive correlations with the upregulated candidate genes in the AH group and negative correlations with those in the CS group.

To explore the relationship between host genes and microorganisms, we further analyzed the association between the differential microbes and candidate genes. Spearman analysis revealed significant correlations between the candidate genes and host microbes. In rumen (Figure 9C), of these 16 candidate genes, 8 were significantly associated with *Oscillospiraceae* (ASV136), and 11 different candidate genes were each significantly associated with either *MVP-15* (ASV887), *Prevotellaceae* (ASV98) or *Rikenellaceae* (ASV131). In jejunum (Figure 9D), 8, 8, 4, 3, and 2 candidate genes were significantly positively correlated with *Lachnospiraceae* (ASV775), *Muribaculaceae* (ASV627), *Ruminococcaceae* (ASV1040), *Enterobacteriaceae* (ASV19), and *Rikenellaceae* (ASV528), respectively.

### 3.11. Effect of the Maternal Diet on Doe’s Milk Metabolome

**Figure 10:**
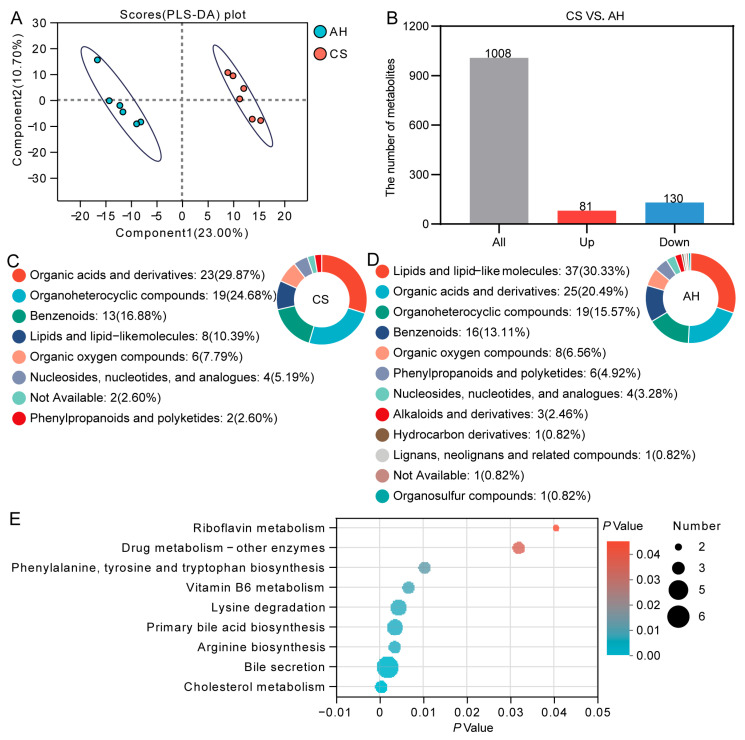
Effect of the maternal diet on doe’s milk metabolome. (**A**) PLS-DA plot of milk metabolites. (**B**) Differential metabolite quantity statistic. Classification of upregulated metabolites in CS group (**C**) and AH group (**D**) based on HMDB database annotation. (**E**) KEGG functional enrichment map of differential metabolites. CS, corn straw group. AH, alfalfa hay group. Circles, 95% confidence interval. *n* = 6/group.

We conducted a metabolomic analysis of doe milk to determine how it responded to different dietary sources (Figure 10). The PLS-DA revealed a significant distinction between the groups (Figure 10A). A total of 1008 metabolites were identified in the milk (Figure 10B). Compared with the AH group, 81 metabolites were significantly upregulated, while 130 metabolites were significantly downregulated in the CS group (VIP > 1, *p* < 0.05; Figure 10B). We further classified these differential metabolites using the HMDB database. In the CS group, the 81 upregulated metabolites were primarily classified into 23 organic acids and derivatives, 19 organoheterocyclic compounds, 13 Benzenoids, and 8 lipids and lipid-like molecules (Figure 10C). In the AH group, 130 downregulated metabolites included 37 lipids and lipid-like molecules, 25 organic acids and derivatives, 19 organoheterocyclic compounds, and 16 benzenoids (Figure 10D).

Subsequently, 211 differential metabolites were subjected to KEGG enrichment analysis (Figure 10E). Nine metabolic pathways implicated in lipid metabolism (cholesterol metabolism, bile secretion, primary bile acid biosynthesis), amino acid metabolism (arginine biosynthesis, lysine degradation, phenylalanine, tyrosine, and tryptophan biosynthesis), cofactors and vitamins metabolism (vitamin B6 metabolism, riboflavin metabolism) and drug metabolism showed significant differences between the AH and CS group. A total of 25 metabolites were implicated in these pathways (Table S5). Notably, 13 metabolites were upregulated in the AH group, involved in lipid metabolism, lysine degradation, and the biosynthesis of phenylalanine, tyrosine, and tryptophan. 12 metabolites were upregulated in the CS group, related to vitamin metabolism, arginine biosynthesis, and drug metabolism.

### 3.12. Maternal Diet Reshaped Offspring Gastrointestinal Microbial Composition via Alters Milk Metabolites

**Figure 11:**
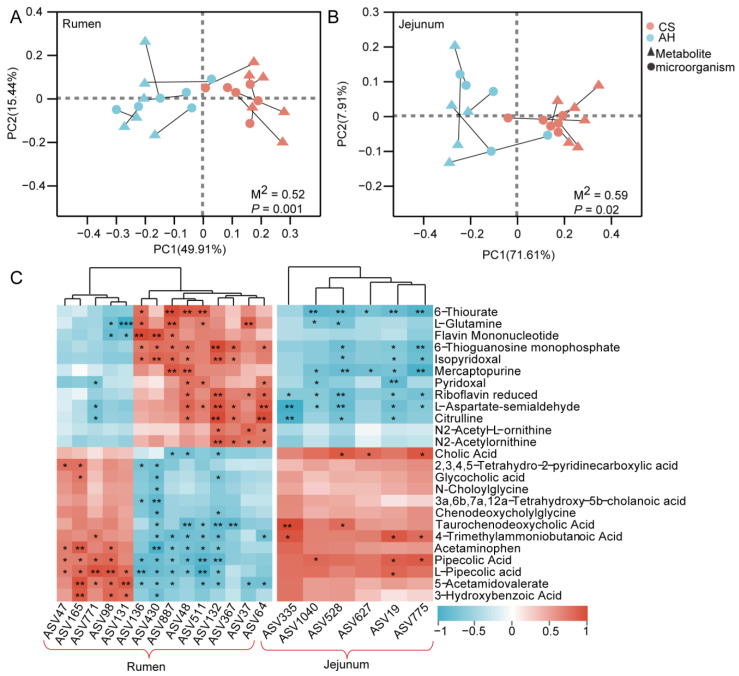
Maternal diet reshaped offspring gastrointestinal microbial composition via altered milk metabolites. Procrustes analysis for correlations between differential metabolites and rumen (**A**) and jejunum (**B**) microbiota in kids. (**C**) Spearman correlation analysis of differential milk metabolites in does and rumen and jejunum microbiota in kids. * *p*  <  0.05, ** *p*  <  0.01. *** *p*  <  0.001. *n* = 12/group.

To study the effect of milk metabolites on the GI microbiome of kids, Procrustes analysis was performed. The result shows that differential metabolites were significantly associated with the rumen (*p*  =  0.001, M^2^ = 0.52) and jejunum (*p*  =  0.02, M^2^ = 0.59) microbiota of the kids (Figure 11A,B). Furthermore, Spearman correlation analysis was conducted to determine the relationship between 25 metabolites and the GI signature microbiome of the kids (Figure 11C). In the CS group, a total of 49 metabolite–microbe associations were observed, involving 12 metabolites and 9 rumen microbial markers. Among these, 6-thiourate, isopyridoxal and L-aspartate-semialdehyde were the top three metabolites associated with microbial markers. In contrast, metabolites related to lysine degradation (4-trimethylammoniobutanoic acid, 5-acetamidovalerate, pipecolic acid, L-pipecolic acid, 2,3,4,5-tetrahydro-2-pyridinecarboxylic acid), phenylalanine, tyrosine, and tryptophan biosynthesis (3-hydroxybenzoic acid) and lipid metabolism (glycocholic acid, acetaminophen) exhibited significant positive correlations with rumen microbiota markers (ASV47, ASV165, ASV771, ASV98, and ASV131) in the AH group. Similarly, in the AH group, 12 metabolite–microbe associations were significant in the jejunum, predominantly related to lipid metabolism (taurochenodeoxycholic acid, cholic acid) and lysine degradation (4-trimethylammoniobutanoic acid, pipecolic acid, L-pipecolic acid). These findings suggest that maternal milk is a substantial factor in modulating the GI microbiota structure of kids.

## 4. Discussion

During early development, young animals rely on milk as their main source of nourishment [28]. Concurrently, they also acquire their microbiome from their mothers via contact and breastfeeding [29]. Maternal nutritional status and microbial composition are theorized to significantly influence the development of progeny tissues and microbiomes. Our study found that the does in the CS group had significantly lower dry matter intake during gestation and lactation compared with those in the AH group. Goat dry matter intake is influenced by the quality of roughage, with high ADF content in feed negatively impacting intake [30]. Thus, the reduced feed intake observed in the CS group may be attributed to the higher ADF content in the diet of does. Additionally, this decrease in feed intake may be attributed to the high fiber content and relatively low bulk density of straw, which create a filling effect in the GI system, reducing feed consumption [31]. In this study, although the does in the AH group exhibited higher feed intake during both gestation and lactation, they demonstrated a similar tendency to gain weight during pregnancy as the does in the CS group. During lactation, the body weights of both the does and their kids in the AH group were numerically higher than those in the CS group. However, statistical analysis revealed no significant weight difference between the two groups. This non-significant result may be due to the uneven distribution of weight data. Further analysis indicated that the does in the CS group had higher digestibility of NDF and ADF during gestation compared with those in the AH group, with NDF digestibility remaining superior during lactation. NDF and ADF are key indicators of indigestible components in feed, such as cellulose and lignin [32]. The higher digestibility of these components in the CS group suggests that the rumen microorganisms of CS-fed does may be more efficient at utilizing these components in roughage [33]. Therefore, although feed intake in the CS group was significantly lower than that in the AH group during both gestation and lactation, the lack of a significant difference in the body weight of both does and kids between the two groups may be explained by these factors.

The GI is crucial for digestion and nutrient absorption, and its early development is essential for animals to maximize their growth potential [10]. Therefore, we further assessed the impact of different maternal diets on goat kid GI development in four aspects: GI morphology, immune function, metabolism, and barrier structure. The morphology of the GI tract, including rumen papillae morphology, intestine villi height, crypt depth, and VH/CD ratio, determines the efficiency of nutrient digestion and absorption [34]. Moreover, the thickness of the muscle layer plays a significant role in rumination and intestinal peristalsis, ensuring efficient digestion. In our study, kids in the CS group showed a higher thickness of the rumen muscle compared with the AH group, which may herald a better digestive ability after the kids have ingested solid feed. In contrast, AH kids exhibited a higher rumen papillae width and a higher VH/CD ratio in the jejunum, suggesting better absorption and metabolism.

The efficient digestion and absorption of nutrients in animals are closely linked to the support provided by the immune system. The immune response is important for fighting pathogens and maintaining the functionality of tissues and organs [35,36]. Cytokines and immunoglobulins are essential components of the immune regulatory network; cytokines such as IL-6 and TNF-α are pivotal in the inflammatory response, and their expression levels serve as reliable indicators of the intensity and status of the immune reaction to external threats or internal damage [37]. In contrast, the anti-inflammatory cytokine IL-10 helps maintain immune homeostasis by inhibiting the excessive activation of immune cells, thus promoting the resolution of inflammation and maintaining balance within the immune system [38]. Immunoglobulins, including IgG, IgM, and SIgA, are critical effectors in the humoral immune response. They not only defend against infections by specifically binding to and neutralizing pathogens, but also modulate immune cell function through interactions with receptors on their surfaces [39]. This dual functionality of immunoglobulins contributes to the precise regulation of the immune response, ensuring both effective pathogen defense and the prevention of excessive immune activation. In this study, compared with the CS group, kids from AH-fed does exhibited significant increases in rumen IL-10 levels, as well as elevated jejunal levels of SIgA, IgG, and IL-10. This finding suggests that feeding AH to does has a beneficial effect on the immune status of their offspring. The immune system is heavily influenced by microbiome signals, and a diverse core of bacterial species may contribute to the development of more complex immune functions, thereby maintaining stability and balance between the microbiome and the host’s immune system [40,41]. Luo et al. found that the core microbiota in the rumen and feces of goats participate in the production of host immune-related factors such as IgA, IL-2, and IL-6 [42]. Jiao et al. demonstrated that increased ruminal microbial density in goat kids can activate the TLR signaling pathway in the ruminal epithelium, initiating an immune response [43]. Immunomodulators such as IL-10, SIgA, and IgG have been shown to correlate with the composition and richness of host microbes [44,45]. Therefore, the increased levels of GI immune factors observed in kids from the AH group in this study are likely a result of increased microbial richness in the GI tract. Additionally, maternal immune factors have been shown to transfer to offspring, modulating immune development [46]. Moreover, the regulatory role of maternal immune factors on offspring immune development is influenced by the maternal diet [47]. It has been reported that the maternal AH diet boosts the levels of the anti-inflammatory cytokine IL-10, while concurrently reducing pro-inflammatory cytokines IL-6 and TNF-α in both maternal and offspring serum [48]. Therefore, the enhanced immune competence observed in AH-group kids in this study may be related to AH, promoting immune development in the offspring by improving the immune status of the does.

The GI epithelial barrier is a critical defense mechanism, ensuring the integrity of GI structures, facilitating efficient nutrient absorption, and maintaining immune homeostasis. The barrier function of young animals is substantially affected by maternal dietary components, particularly the source of dietary fiber in the feed [8,49]. Our results also support this view, as we found that feeding specific diets, such as AH, to does markedly increased the expression of ZO-1 and MUC2 in the jejunum of their offspring. ZO-1 is a key molecule within tight junction complexes, connecting transmembrane tight junction proteins to the cytoskeleton and playing an essential role in shaping and maintaining tight junction structures and functions [50]. Tight junctions interact with various GI cells, including epithelial, immune, and goblet cells, to facilitate substance transport and provide mechanical support [51]. The intestinal mucus barrier is an important physical barrier for defense against pathogens and is mainly composed of MUC2 mucins secreted by goblet cells [52]. The mucous layer encapsulates and limits bacterial activity while screening and blocking the transepithelial diffusion of metabolites or toxins, thus protecting the host from potential pathogens [53]. In conclusion, our study elucidates the significance of the maternal diet in shaping the development and function of the gut in offspring, as evidenced by changes in barrier-associated proteins.

To further investigate how dam diet affects offspring GI development and function, RNA-seq was performed on rumen and jejunum tissues of kids. In this study, significant alterations in gene expression were observed in the GI tissues of the kids due to varying maternal dietary sources. GO-term enrichment analysis revealed that the cell and structural development processes were more pronounced in the rumen of kids from the CS group. In contrast, immune response and response to stimulus process were more enriched in the AH group. Our findings on rumen morphology and immune factors in the kids provide additional support for the GO enrichment. Specifically, we observed that the rumen of kids in the AH group had elevated IL-10 levels, while those in the CS group had thicker rumen muscle layers, suggesting that the different diets of the does significantly impacted the rumen development of suckling kids at the gene transcriptional level. Furthermore, it has been reported that immune activation alters energy distribution within an animal’s body, with partial energy being diverted towards the immune response, potentially influencing animal development [54,55]. Consequently, the AH-group kids exhibited a relatively lower thickness of the rumen muscle layer, likely due to the enhanced immune activity in the rumen. In the jejunum of the kids, DEG were highly enriched in GO terms associated with the regulation of transferase activity. Transferases catalyze the transfer of functional groups from one molecule to another, thereby participating in the modulation of host immunity, metabolism, and barrier function [56]. The above results demonstrate that maternal dietary sources are linked to changes in the gut function of the offspring. Similar to our results, research conducted by Penagaricano et al. revealed that, while maintaining the same energy supply for ewes, fetuses from AH-fed ewes exhibited higher energy metabolic activity compared with the control group (corn-based feed) [57]. Furthermore, AH-fed ewes significantly promoted the upregulation of genes associated with muscle and fat tissue development in their fetuses, indicating that different feed sources for ewes not only affected the overall energy utilization pattern of the fetuses, but also specifically regulated the gene expression profile related to tissue growth and differentiation [57].

Additionally, in this study, KEGG analysis revealed that the DEGs in the jejunum were predominantly linked to oxidative phosphorylation, thermogenesis, and the ErbB signaling pathway, with these DEGs exhibiting heightened expression in kids from AH-fed does. The marked enhancement of the oxidative phosphorylation process in intestinal tissue is associated with an increased capacity for energy generation, which is indispensable for maintaining the normal functioning of the intestinal system [58,59]. The intestinal epithelium possesses an extraordinary capacity for self-renewal and regeneration, relying on the continuous proliferation and differentiation of stem cells located at the base of the intestinal crypts [60]. Intestinal epithelial cells undergo an ordered migration and maturation along the crypt–villus axis, replacing senescent cells, ensuring continuous renewal of the intestinal epithelium and maintaining the integrity of the intestinal epithelial barrier [61]. Oxidative phosphorylation is crucial for the regeneration of intestinal epithelial cells. As intestinal epithelial cells differentiate and mature, their energy requirements increase. Oxidative phosphorylation, the primary route of ATP production, directly supports the energy supply to the gut, thus enhancing the regenerative capacity of epithelial cells and maintaining the effectiveness of the intestinal barrier function [62,63]. Therefore, the significant enhancement of the oxidative phosphorylation process in the kid jejunum from the AH group may be responsible for the increased VH/CD ratio. Simultaneously, the ErbB signaling pathway mediates cell proliferation and differentiation [64]. Thus, the increased expression of genes linked to the ErbB pathway in AH kids might also be responsible for the enhanced regeneration of the intestinal epithelium.

This study demonstrates that the maternal diet significantly impacts the development and function of the GI tract in kids. However, the specific mechanisms by which different maternal diets affect the offspring GI system remain unclear. It is essential to recognize the crucial role that microorganisms play in the development and functionality of the GI system. These microorganisms interact closely with mucosal cells, immune cells, and neuronal terminals, and actively participate in and profoundly influence the development and functional establishment of host organs [65,66]. Furthermore, microorganisms directly communicate with immune cells to precisely regulate immune response stability and form complex metabolic networks with the host, which significantly affects the host’s metabolic functioning [66,67]. Thus, we speculate that microorganisms play a crucial role in in mediating the impact of the maternal diet on the development and function of the offspring GI tract. The NMDS analysis showed that different maternal feed sources resulted in a clear separation of GI microbiota composition between AH and CS group kids. LEfSe analysis identified 20 different bacterial ASVs that contribute to this separation. Subsequently, we conducted a correlation analysis to investigate the relationship between the DEGs found in significantly enriched pathways and morphological indicators, as well as the significantly differential taxa. Our analysis revealed a significant positive correlation between rumen muscle thickness and the differential genes *RRES2*, *FHOD3*, *EPB41L3*, and *THBS1*. Furthermore, several microbial biomarkers (ASV511, ASV887, ASV37, ASV132, ASV367, and ASV136) exhibited a positive correlation with the aforementioned differential genes. We also found that the width of ruminal papillae was positively correlated with the differential genes *C3* and *NOD2*. In addition, the microbial biomarkers ASV165, ASV131, and ASV98 showed significant positive correlations with both *C3* and *NOD2*. Additionally, 12 DEGs were found to have a positive correlation with 5 microbial biomarkers and 10 intestinal phenotypic indicators in the kid intestines.

Based on the above information, the observed changes in GI phenotypic indicators in this study may be attributed to microbial biomarkers that regulated host gene expression. Previous studies have also shown that microbial communities can influence host performance by modulating gene expression in the host [68]. For instance, during the pre-weaning stage, specific microorganisms in the goat kid rumen are linked to the regulation of adhesion mechanisms in ruminal cells and epidermal development processes. Furthermore, the interaction between the goat transcriptome and microbiome plays a crucial role in maintaining ruminal pH homeostasis, regulating nitrogen metabolism, and modulating immune responses [6]. Similarly, Chen et al. found that specific ruminal microorganisms, such as *Alloprevotella* and the *Oscillospiraceae NK4A214* group, may play a pivotal role in enhancing ruminal epithelial barrier function and facilitating VFA transport by regulating the expression of molecules such as *MCT-4*, *NHE-2*, *NHE-3*, and *Occludin-1* on the ruminal epithelium [7]. Additionally, VFAs produced by certain microbes are closely linked to the expression of genes associated with rumen epithelial development, carbohydrate metabolism, membrane transport, and tight junctions in young ruminants [69]. VFAs regulate host physiological functions by providing energy, modulating lipid metabolism, and maintaining the balance of GI microbiota [70]. In this study, kids in the AH group exhibited elevated jejunal acetate and TVFA levels. Furthermore, significantly enriched biomarker taxa *Enterobacteriaceae* (ASV19), *Ruminococcaceae* (ASV1040), *Rikenellaceae* (ASV528), *Muribaculaceae* (ASV627), and *Lachnospiraceae* (ASV775) in the AH group are major producers of VFAs. Thus, the improved gut development of kids from AH-fed does may be due to an increased concentration of VFA resulting from microbial fermentation.

Notably, the marker microorganisms we screened in the rumen and jejunum of goat kids in both groups were also enriched in the rumen or milk of the does. Meanwhile, we observed a significant correlation between the GI microbiome of the kids and the rumen and milk microbiome of their mothers. Emerging evidence indicates that microorganisms from maternal sources such as saliva, milk, skin, birth canal, and rumen can be transmitted vertically to offspring, contributing to the early colonization of their microbiomes and influencing the development and functional establishment of the GI system [71,72]. In this study, 61–68% of the rumen microorganisms found in does were also present in the rumen of the kids, while 80–87% of the milk microorganisms were found in kid intestines. Karkman et al. found that approximately 76% of milk microbes are found in the gut of infants [73]. The maternal microbes colonizing the offspring’s gut can affect gut development by reprogramming the transcriptional profile, promoting the maturation of the immune system and encoding the expression of tight junction proteins, thereby altering gut morphology [74,75]. Jin et al. discovered that the rumen microbiota of dams can influence the composition of their offspring’s rumen microbiota and also impact their growth performance [72]. These findings, along with those of the present study, suggest that the maternal microbiome transferred to the offspring during lactation interacts with the host to drive the development and functional establishment of the GI system [76]. Furthermore, the correlation analyses conducted in this study indicate that milk metabolites also play a crucial role in shaping the gut microbiome of the kids. We found that the most significantly enriched KEGG pathways of differential metabolites were lipid metabolism, amino acid metabolism, cofactor and vitamin metabolism, and drug metabolism. Notably, enriched metabolites in metabolic pathways, such as bile acids, amino acids and their derivatives, and vitamins and their derivatives, were significantly associated with the gut microbiome of the kids. Bile acids are crucial for shaping the animal microbiome by promoting the growth of bile acid-metabolizing bacteria, inhibiting sensitive bacteria, and regulating the gut microbiome composition through the activation of innate immune genes in the small intestine [77,78]. Amino acids provide a nitrogen source for microorganisms, facilitating microbial growth [79,80]. Vitamins act as cofactors or coenzymes to participate in various metabolic reactions of microorganisms, thereby affecting their growth and activity [81,82]. Thus, the effect of maternal diet on the offspring microbiota may be attributed to the transfer of milk metabolites, promoting microbial-dependent growth [83].

## 5. Conclusions

Our findings suggest that does regulate the establishment of the kid GI microbiome through the vertical transmission of rumen microbes, milk microbes and their metabolites. This process positively influences GI development and function. Specifically, the type of roughage provided to does may lead to corresponding divergences in the GI microbiome of suckling kids, accompanied by differences in genes associated with GI immunity, structural development, and energy metabolism, which are important for GI tissue morphogenesis, barrier function enhancement, immune system maturation, and metabolic regulation. Though our study also revealed that the doe AH diet is particularly beneficial for regulating the GI development of offspring, kids from AH-fed or CS-fed does did not develop diarrhea under the current experimental conditions and there was no significant difference in mortality rates between the two groups. The result shows that both dietary choices had a positive effect on the overall health of the kids, provided the nutritional requirements of the does were met.

## Figures and Tables

**Table 1:** Effects of roughage sources on doe apparent nutrient digestibility.

Items	CS	AH	*p*-Value
Gestation			
Crude protein	68.45 ± 1.03	67.40 ± 1.15	0.39
Crude fat	70.16 ± 1.07	67.25 ± 1.95	0.06
Neutral detergent fiber	68.01 ± 1.16	52.93 ± 5.56	0.01
Acid detergent fiber	61.73 ± 3.23	50.34 ± 0.59	0.03
Lactation			
Crude protein	71.45 ± 2.33	68.87 ± 1.58	0.26
Crude fat	71.90 ± 1.28	70.32 ± 3.00	0.53
Neutral detergent fiber	67.99 ± 1.93	57.66 ± 1.30	<0.01
Acid detergent fiber	58.87 ± 3.22	54.72 ± 3.13	0.26

CS, corn straw diet; AH, alfalfa hay diet; *n* = 12/group.

## Data Availability

The data sets were deposited in the NCBI SRA: PRJNA1172082.

## Supplementary Materials

The following supporting information can be downloaded at www.mdpi.com/article/10.3390/ani15030393/s1, Table S1. Composition and nutrient levels of experimental diets (DM basis%). Table S2. Significantly enriched pathways and gene sets. Table S3. Relative abundance on phylum level in different groups (%). Table S4. Relative abundance on genus level in different groups (%). Figure S1. Procrustes analysis for correlation between doe differential microbes and kid differential microbes. Table S5. Significantly enriched KEGG pathway of differential metabolites.
