# Supplementary material for: Maternal Roughage Sources Influence the Gastrointestinal Development of Goat Kids by Modulating the Colonization of Gastrointestinal Microbiota
Source: Animals (Basel). 2025 Jan 30;15(3):393. doi: 10.3390/ani15030393 (PMC11815875; doi:10.3390/ani15030393)
Supplement: Supplementary file 1 [file animals-15-00393-s001.zip › animals-3396297-supplementary.pdf]

Supplementary Table S1. Composition and nutrient levels of experimental diets (air-dry basis) %

Items	CS	AH
Ingredients		
Corn	20.00	33.00
Soybean meal	19.00	-
Corn straw	50.00	-
Alfalfa hay	-	50.00
Wheat hay	1.00	5.00
concentrate supplement[a]	8.50	8.50
Zeoilte powder	-	2.00
$NaHCO_3$	0.25	0.25
Salt	0.25	0.25
Premix[b]	1.00	1.00
Total	100.00	100.00
Nutrient Levels[c]		
ME, MJ/kg	9.21	9.20
CP	13.97	13.97
EE	3.60	4.12
Ash	5.61	5.67
NDF	43.81	29.07
ADF	25.70	19.17

[a]The ewe concentrate supplement containing corn, soybean meal, cottonseed meal, Corn husk, urea Calcium hydrogen phosphate, NaCl and stone powder was purchased from Zhengda (Zhengda, Hohhot, China).

[b]Provided per kilogram of premix: vitamin A 300000 IU/kg; vitamin D3 85000 IU/kg; vitamin E 1600 IU/kg; vitamin B1 20 mg/kg; vitamin B2 55 mg/kg; vitamin B6 12 mg/kg; niacin 240 mg/kg; pantothenate 120 mg/kg; folic acid 9 mg/kg; biotin 3 mg/kg; Fe 600 mg/kg; Cu 200 mg/kg; Zn 1200 mg/kg; Ca 300 mg/kg; P 25 mg/kg.

[c]ME was calculated value, while others were all measured values.

Supplementary Table S2. Significantly enriched pathways and gene sets

GO/KEGG ID	Description	*Padj*	Gene Name
	Rumen		
GO:0010927	cellular component assembly involved in morphogenesis	0.08	KLHL41/EPB41L3/FHOD3/ACTN2/CFL2
GO:0002504	antigen processing and presentation of peptide or polysaccharide antigen via MHC class II	0.08	HLA-DRA/CD74/THBS1
GO:0030239	myofibril assembly	0.08	KLHL41/FHOD3/ACTN2/CFL2
GO:0006954	inflammatory response	0.08	C3/RARRES2/SERPINE1/NOD2/IL1RL2/DUSP10/TSPAN2/THBS1/S100A8
GO:0032103	positive regulation of response to external stimulus	0.08	C3/RARRES2/CD74/SERPINE1/NOD2/THBS1/S100A8
	Jejunum		
GO:0051338	regulation of transferase activity	0.05	HRAS/EREG/PTK6/GADD45A/UBE2S/EPM2AIP1/APC/BAD/PHPT1/PIN1
chx05210	Colorectal cancer	<0.01	AREG/HRAS/EREG/GADD45A/APC/BAD
chx05213	Endometrial cancer	0.02	HRAS/GADD45A/APC/BAD
chx00190	Oxidative phosphorylation	0.02	NDUFS6/COX17/NDUFA13
chx04714	Thermogenesis	0.05	/HRAS/NDUFS6/COX17/NDUFA13
chx04012	ErbB signaling pathway	0.05	AREG/HRAS/EREG/BAD

Supplementary Table S3. Relative abundance on phylum level in different groups (%)

**Phylum**	**CS group**	**AH group**
Rumen phylum		
Bacteroidota	62.47	69.10
Firmicutes	23.71	21.87
Proteobacteria	6.84	2.86
Elusimicrobiota	2.06	0.09
Verrucomicrobiota	1.09	2.43
other	3.83	3.65
Jejunum phylum		
Firmicutes	62.75	70.08
Proteobacteria	14.02	17.79
Bacteroidota	2.80	6.28
Actinobacteriota	3.49	2.52
Cyanobacteria	0.72	0.79
Others	16.23	2.54

Supplementary Table S4. Relative abundance on genus level in different groups (%)

Genus	CS group	AH group
Rumen genus		
F082	23.59	18.11
SP3-e08	8.47	12.34
Muribaculaceae	1.91	7.31
Rikenellaceae_RC9_gut_group	3.56	5.69
Alloprevotella	5.58	1.93
NK4A214_group	1.38	1.61
Christensenellaceae_R-7_group	1.67	1.63
Prevotellaceae_UCG-003	2.32	3.41
Prevotella	3.57	3.34
Prevotellaceae_UCG-004	1.39	1.63
other	46.55	43.00
Jejunum genus		
Lactobacillus	39.53	42.08
Ralstonia	2.87	0.96
Moraxella	1.19	2.50
Neisseria	0.84	0.54
Bacillus	1.15	1.29
Alysiella	0.82	1.04
Clostridium_sensu_stricto_1	1.42	0.21
Achromobacter	0.69	0.34
Chloroplast	0.64	0.78
Streptococcus	0.72	1.69
other	50.12	48.55

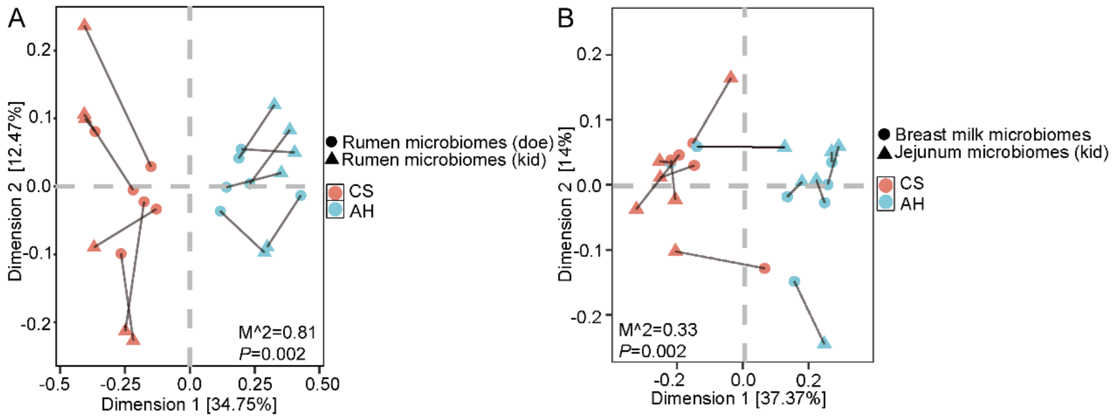

Figure S1. Procrustes analysis for correlation between doe differential microbes and kid differential microbes. (**A**) Correlation between rumen microbiota in does and kids. (**B**) Correlation between milk microbiota in ewes and jejunum microbiota in kids. CS, corn straw group. AH, Alfalfa hay group.

Supplementary Table S5. Significantly enriched KEGG pathway of differential metabolites

KEGG pathway ID	KEGG pathway description	Metabolite	*P* value	*Padj*
map00740	Riboflavin metabolism	Flavin Mononucleotide/Riboflavin reduced	0.04	0.26
map00750	Vitamin B6 metabolism	Isopyridoxal/L-Glutamine/Pyridoxal	0.01	0.06
map00400	Phenylalanine, tyrosine and tryptophan biosynthesis	3a,6b,7a,12a-Tetrahydroxy-5b-cholanoic acid/L-Aspartate-semialdehyde/3-Hydroxybenzoic Acid	0.01	0.08
map00983	Drug metabolism - other enzymes	6-Thioguanosine monophosphate/6-Thiourate/Mercaptopurine	0.03	0.23
map04979	Cholesterol metabolism	Taurochenodeoxycholic Acid/Chenodeoxycholylglycine/Glycocholic acid/N-Choloylglycine	<0.01	0.01
map00220	Arginine biosynthesis	N2-Acetyl-L-ornithine/Citrulline/N2-Acetylornithine/L-Glutamine	<0.01	0.06
map00120	Primary bile acid biosynthesis	Taurochenodeoxycholic Acid/Cholic Acid/Chenodeoxycholylglycine/Glycocholic acid/N-Choloylglycine	<0.01	0.05
map00310	Lysine degradation	4-Trimethylammoniobutanoic Acid/5-Acetamidovalerate/Pipecolic Acid/L-Pipecolic acid/2,3,4,5-Tetrahydro-2-pyridinecarboxylic acid	<0.01	0.05
map04976	Bile secretion	Taurochenodeoxycholic Acid/Cholic Acid/Chenodeoxycholylglycine/Glycocholic acid/Acetaminophen/Mercaptopurine/N-Choloylglycine	<0.01	0.05
